# Mating system and population structure in the natural distribution of *Toona ciliata* (Meliaceae) in South China

**DOI:** 10.1038/s41598-020-74123-8

**Published:** 2020-10-12

**Authors:** Wei Zhou, Xin-Xin Zhang, Ying Ren, Pei Li, Xiao-Yang Chen, Xin-Sheng Hu

**Affiliations:** 1 College of Forestry and Landscape Architecture, South China Agricultural University, Guangdong, 510642 China; 2 Guangdong Key Laboratory for Innovative Development and Utilization of Forest Plant Germplasm, South China Agricultural University, Guangdong, 510642 China

**Keywords:** Population genetics, Population genetics

## Abstract

Most initially perfect flowers of *Toona ciliata* Roem subsequently develop into functionally unisexual flowers and their relative positions in the same inflorescence could enhance the outcrossing system in this species. Here we investigated the mating system of this species. We used eight nuclear microsatellite markers and investigated the progeny of 125 mother trees from six populations naturally distributed in South China, with sample sizes ranging from 64 to 300 seeds. The multilocus outcrossing rate was 0.970 ± 0.063, and the single locus outcrossing rate was 0.859 ± 0.106, indicating the pattern of predominant outcrossing. Selfing was present in one population, but biparental inbreeding occurred in five populations. Inbreeding was absent in maternal parents, and correlations of selfing among families or among loci were generally insignificant. Positive correlation of paternity at multiple loci was significant in four populations, but was not consistent with the results at single loci. Population substructure occurred in male similarity between outcrosses only in one population. Population genetic differentaitaion was significant (*F*_*st*_ = 34.5%) and the effects of isolation-by-distance at the eight loci were significant among the six populations. These results provide evidence that self-comptability and inbreeding naturally occur in *T. ciliata* and indicate that inbreeding avoidance is necessary during genetic improvement and breeding of this endangered tree species.

## Introduction

The flower structure of *Toona ciliata* Roem (Meliaceae) is characterized by flowers of about 5 mm long. There are short pedicels of 1–2 mm long, five short calyxes, five white and oblong petals each 4–5 mm long, five stamens that are about as long as the petals, filaments that are sparsely pilose, elliptic anthers, an ovary that is densely covered with long hard hairs and contains 8–10 ovules per locule, a glabrous style, and discoid stigmas with five fine lines^1^. The flowers in the bud stage are perfect and hermaphrodite in the family of Meliaceae. Owing to the effects of unknown genetic or environmental factors, most floral development exhibits functionally male or female floewers although both anthers and pistils are present in each flower^2,3^. Sterile ovaries in functionally male flowers are formed and would not develop further. Stamens with anthers in functionally female flowers seem not to produce fertile pollen. This is an alternative to those patterns that avoid selfing and inbreeding by forming sexually diecious plants or employing self-incompatible genes. However, it is not clear whether such floral development is ubiquitous or not in genus *Toona* and other genera (e.g., *Cedrela* and *Swietenia*) of the Meliaceae^2^, and the flowers that remain perfect cannot be excluded. The functionally male and female flowers are disproportionally mixed and distributed in different positions in the same inflorescence. The central flower in a cymule is female while the lateral flowers are male^2,3^. Most individuals are sexually monoecious rather than dioecious. Thus, it is of interest to clarify the uncertainty of the mating system that *T. ciliata* possesses.

Preliminary inferences on the outcrossing rate of this species could be inferred from a recent study on population structure^4^. Based on the provenance populations of mixed seeds from multiple indidvisuals, the estimate of inbreeding coefficients was generally negative (*F*_is_ < 0), implying an outcrossing system under an equilibrium between selfing and outcrossing where the outcrossing rate is $t=(1-{F}_{is})/(1+{F}_{is})$^5^. However, more accurate estimate of the outcrossing rate or the selfing rate (α = 1 − *t*) remains lacking based on the pedigree of famility structure, where the maximum likelihood estimate of outcrossing rate can be derived under disequilibrium between selfing and outcrossing^6^.

Scoring mating system of *T. ciliata* is of particular significance to its genetic conservation or sustainable management. This species is classified as an endangered species in China^7^ although it is not in the”Red lists” of the species at risk by the International Union for Conservation of Nature and and Natural Resources (IUCN). The importance of this timber tree species is due to its straight stem form and quality red wood for furniture in China. However, the low natural regeneration and over-cutting enhanced a substantial decline in population density^8,9^. This subsequently leads to the influences on mating system due to population demography and changes genetic variation within and between populations. So far, several programs have been set up to promote artificial plantations and genetic breeding, including provenance trials^10^ and the ongoing breeding program for insect-resistance. The critical issue is that the initial breeding programs were based on open-pollinated seeds derived from natural populations with unknown mating systems^10,11^, which could yield biased estimates of genetic parameters and evaluation of progeny performance^12^.

**Figure 1:**
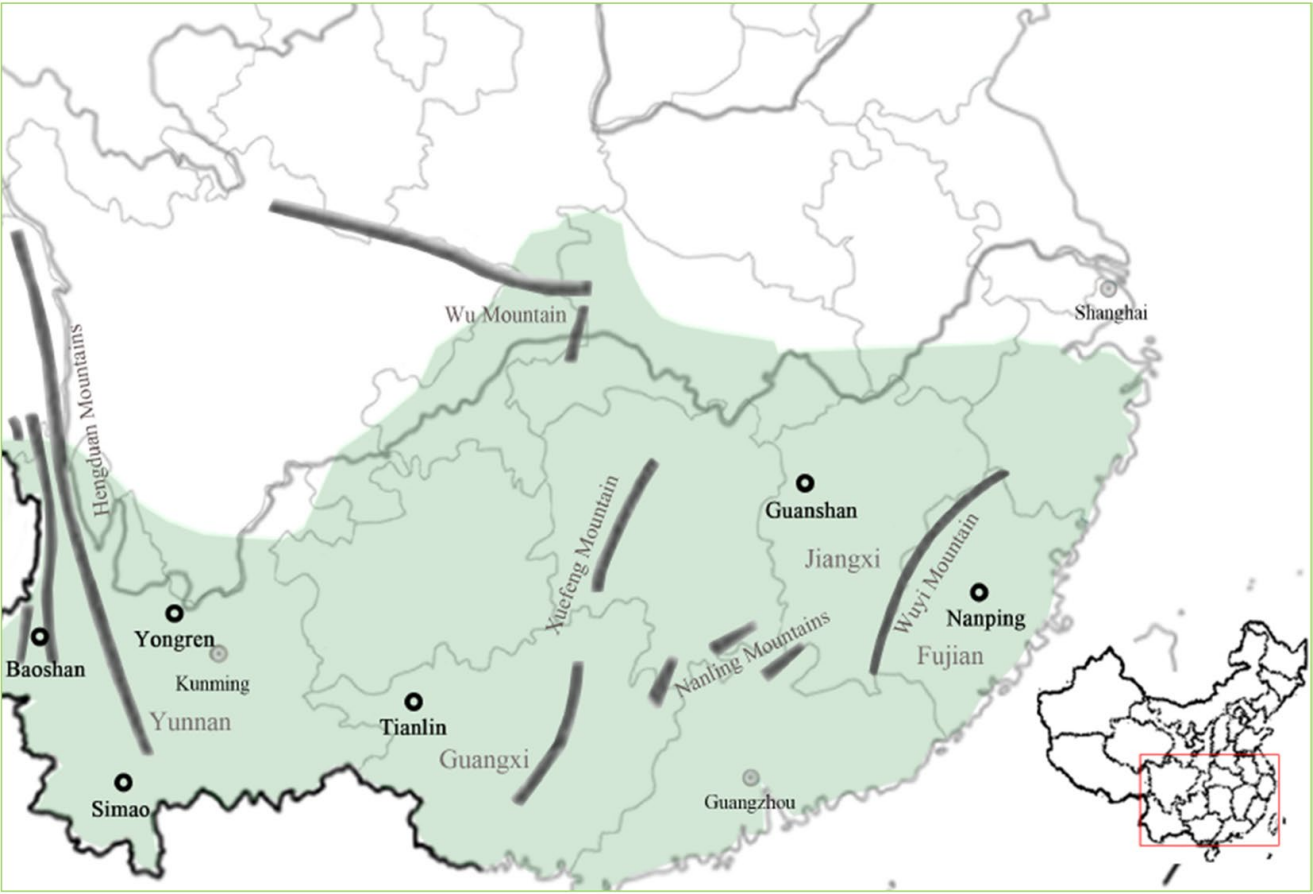
Locations of six populations of *Toona ciliata* for mating system analysis (Baoshan, Simao, Yongren, Tianlin, Guanshan and Nanping). The approximate natural geographical distribution of this species (in green) was plotted using Adobe Photoshop 2018 according to the distribution of 234 specimens available from https://www.cvh.ac.cn/ and a reference to Chen et al.^1^. The natural distribution of *T. ciliata* covers the regions of eastern, southern, central and southwest China.

Here we investigated mating system of six natural populations distributed in South China. Previous studies with SRAP (sequence-related amplified polymorphism) and nuclear microsatellites (simple sequence repeats, SSRs) indicated that substantial genetic variation (about 79.26% with SRAP versus 32.4% with SSR) occurred among populations^4,13^. The range-wide distribution of *T. ciliata* was grouped into two clusters, the western and eastern parts. To assess variation of mating system in different regions, six samples were collected from populations in both western and eastern parts of the species distribution (Fig. 1). We used nuclear SSRs because of the attributes of highly informative microsatellite markers for paternity/parentage and mating system analyses^14,15^. SSR primers that were previously screened in *T. ciliata* were used for individual genotyping^4^. Besides, population genetic structure among the six populations was analyzed and then combined with the estimates of mating systems to inform strategies for managing this endangered forest species in China.

## Results

Eight pairs of SSR primers were screened according to the most sequence variability observed in six populations (Table 1). Genotyping data at eight loci are shown in Supplementary Information Table 1. Analysis with Micro-Checker indicated that null alleles at locus TCR20 existed in all six populations, followed by locus TCR17 in three populations (Simao, Yongren and Tianlin), and loci TCR51 (Tianlin and Nanping), TCR83 (Simao and Yongren), and TCR81 (Yongren and Nanping) in two populations. Loci TCR26 (Guanshan) and TCR51 (Nanping) exhibited null alleles in one population. Three loci potentially had mis-scoring of stutters, including TCR18, TCR20 and TCR51 in Nanping (Table 2).

**Table 1:** Primer sequences, repeat motifs, annealing temperature, allele sizes and the number of alleles at eight microsatellite loci for genotyping in six natural populations of *Toona ciliata.*

Locus	Forward/reverse primers (5′–3′)	Repeat motifs	*T*_a_ (°C)	Allele sizes	Number of alleles
TCR17	F: GTGGCGTAACAGACCAAAAC R: CCAGAGATACTCCATTCCAG	(GA) n	58	140–205	14
TCR18	F: GAAACCAGCAGGCAGAGC R: GAAGAAGGGTGAGCGAGA	(AG) n	58	107–210	44
TCR20	F: AAGCCAGTCAGCAACCTA R: GATTAAGTAATATTGGGTGGT	(GA) n	58	152–327	30
TCR26	F: ATGGATGAGTGTGCGATAGG R: TGTGATGTAGGAGTCTGAAC	(TC) n	58	233–271	30
TCR51	F: CAAAGACCAAGATTTGATGC R: ACTATGGGTGGCACAACTAC	(GA) n	56	108–140	5
TCR78	F: GATCTCACCCACTTGAAAAA R: GCTCATATTTGAGAGGCATT	(GA) n (AG) n	58	163–255	24
TCR83	F: GAGATACAGTTGGTGGTTAGAGG R: TCTTCACCTGTTTGCCTCTC	(CT) n	58	183–287	47
TCR122	F: GTGCAGTGTCCATGTTGAAG R: GACATTTTCTCTGCAAGGTCA	(CA) n	56	158–298	14

**Table 2:** Location, sample size, genotyping errors and genetic diversity in six natural populations of *Toona ciliata.*

Populations	Latitude	Longitude	Altitude (m)	Samples	Stuttering loci	Large allele dropout	Loci with null alleles	Observed heterozygosity (*H*_*o*_)	Expected heterozygosity with null alleles (*H*_*e*_)^a^	Mean number of observed alleles per locus
Baoshan, Yunnan	N 24° 59′	E 99° 01′	1401	132 seeds (23 families, 4–6 seeds/family)	No	No	TCR20	0.505 ± 0.266	0.513 ± 0.219	12.750 ± 8.956
Simao, Yunnan	N 22° 46′	E 100° 58′	600	64 seeds (12 families, 4–6 seeds/family)	No	No	TCR17, TCR20, TCR83	0.227 ± 0.273	0.345 ± 0.249	5.500 ± 4.660
Yongren, Yunnan	N 26° 01′	E 101° 40′	1580	90 seeds (16 families, 4–6 seeds/family)	No	No	TCR17, TCR20, TCR83	0.266 ± 0.291	0.371 ± 0.245	7.000 ± 4.986
Tianlin, Guangxi	N 24° 17′	E 106° 13′	1200	119 seeds (20 families, 4–6 seeds/family)	No	No	TCR17, TCR20, TCR51	0.494 ± 0.310	0.603 ± 0.200	12.250 ± 9.285
Guanshan, Jiangxi	N 28° 32′	E 114° 33′	330	138 seeds (24 families, 4–6 seeds/family)	No	No	TCR18, TCR20, TCR26	0.332 ± 0.264	0.451 ± 0.281	7.250 ± 5.444
Nanping, Fujian	N 26° 38′	E 118° 10′	800	300 seeds (30 families, 10 seeds/family)	TCR18, TCR20, TCR51	No	TCR18, TCR20, TCR51, TCR78	0.256 ± 0.243	0.434 ± 0.286	2.875 ± 1.246

^a^The expected heterozygosity was calculated according to the allele frequencies in SI Table 1 where null allele frequencies were estimated if present at a locus.

Allele frequencies were estimated using Genepop V4 for the loci without null alleles^16^, but with Micro-Checker (Oosterhout’s method) for the loci with null alleles for each population (SI Table 2)^17^. Eight SSR loci exhibited a quite high level of variation among populations in heterozygosity and the number of alleles. Tianlin and Baoshan had the highest mean of observed heterozygosity over loci, followed by Guanshan, Yongren, Nanping and Simao. The expected heterozygoity was calculated according to the estimates of allele frequencies (SI Table 2). The expected heoertozygosity (with the null alleles counted) was greater than the observed heterozygosity at each locus, but had the same ranking pattern as the observed heterozygosity among populations. This was slightly different from the pattern of the average number of observed alleles where Nanping (2.875 ± 1.246) and Simao (5.500 ± 4.660) had a fewer alleles (Table 2).

**Figure 2:**
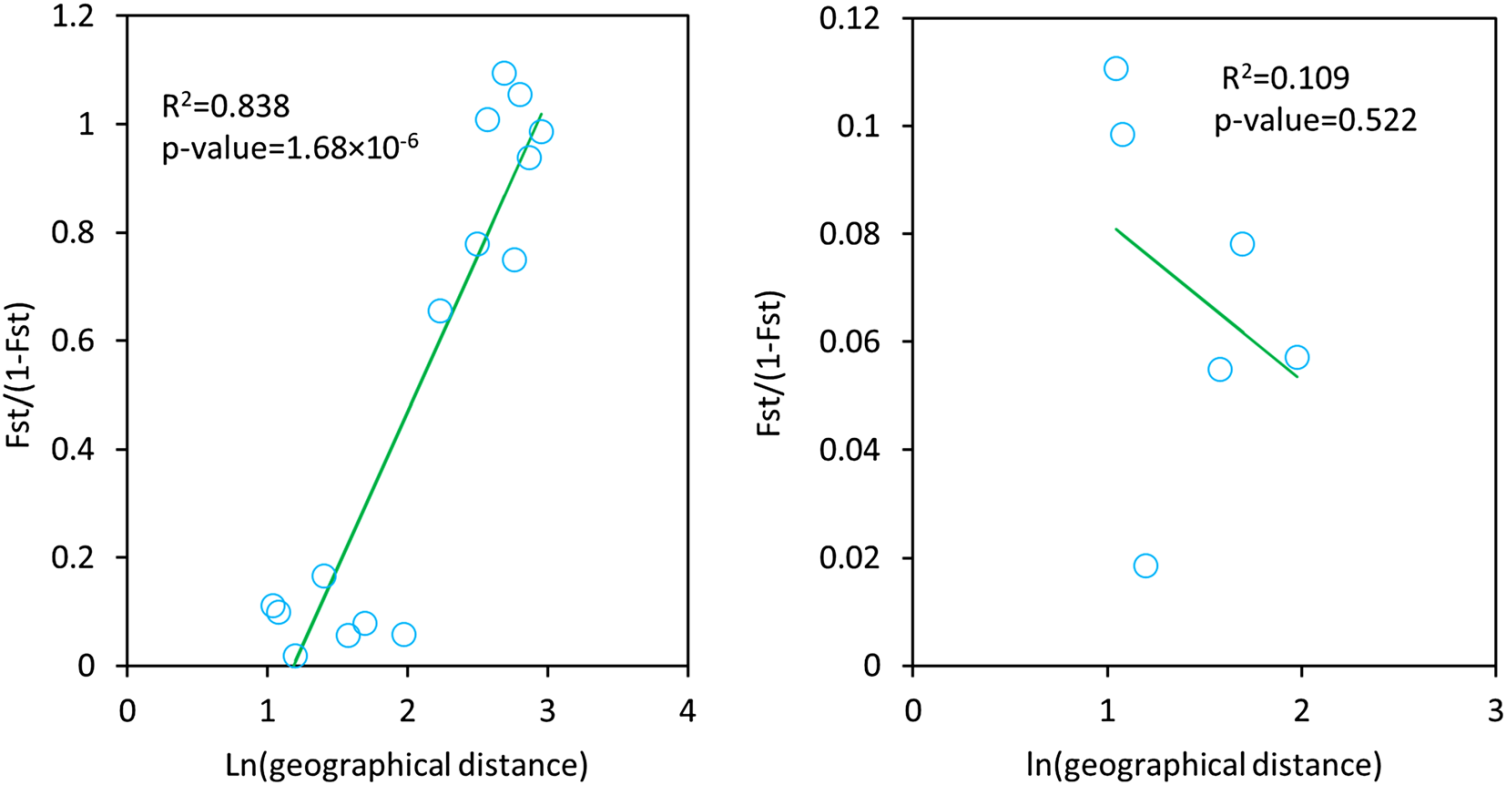
Test of isolation-by-distance (IBD) effects based on multilocus estimates of pairwise *F*_*st*_’s. (**A**) Significant IBD effects existed among the six natural populations, $\frac{{F}_{st}}{1-{F}_{st}}=-0.752+0.7ln(\mathrm{geographical distance})$; (**B**) Insignificant IBD effects occurred among four populations in the western region (without Guanshan and Nanping).

Analysis of population genetic differentaion with Eqs. (2) and (3) was summarized in Table 3. Generally, population genetic differentiation was significant at each locus except TCR51, ranging from *F*_*st*_ = 0.005 at locus TCR51 to 0.599 at locus TCR17. About 34.5% of SSR variation was accounted for by the differences among populations. Significant relationships between *F*_*st*_/(1 − *F*_*st*_) and the logarithmic geographical distance were present at all eight loci (Table 4). However, the analysis with multiple loci also showed a significant relationship between *F*_*st*_/(1 − *F*_*st*_) and the logarithmic geographical distance (p-value = 1.68 × 10^–6^, Fig. 2), indicating the occurrence of isolation-by-distance (IBD) among the six natural populations. When the two populations from the eastern region (Guanshan and Nanping) were not included for analysis, IBD effects were insignificant (Fig. 2). Correlations between *F*_*st*_ and the altitude difference were not significant at both single and multiple loci (Table 3).

**Table 3:** Population genetic differentiation (*F*_*st*_), isolation-by-distance (IBD) and correlations between *F*_*st*_ and altitude differences between populations at eight polymorphic SSR loci in six populations

Locus	*F*_*st*_ (p-value)	*a* (p-value)	*b* (p-value)	R square	$r ({F}_{st}$, altitude)
TCR17	0.599 (0.0000)	− 4.202 (0.021)	3.155 (0.003)	0.647	0.370 (0.262)
TCR18	0.268 (0.0000)	− 0.433 (0.010)	0.367 (8.81 × 10^–5^)	0.706	0.382 (0.160)
TCR20	0.302 (0.0000)	− 0.403 (0.027)	0.394 (1.39 × 10^–4^)	0.685	0.347 (0.205)
TCR26	0.167 (0.0000)	− 0.055 (0.358)	0.102 (0.002)	0.537	0.297 (0.283)
TCR51	0.005 (0.6215)	0.001 (0.767)	0.003 (0.015)	0.403	0.334 (0.244)
TCR78	0.622 (0.0000)	− 4.635 (0.028)	3.497 (0.004)	0.620	0.404 (0.218)
TCR83	0.246 (0.0000)	− 0.255 (0.047)	0.265 (2.44 × 10^–4^)	0.657	0.366 (0.179)
TCR122	0.207 (0.0000)	− 0.090 (0.265)	0.141 (0.002)	0.550	0.287 (0.296)
Over loci	0.345 (0.0000)	− 0.682 (0.0007)	0.576 (1.68 × 10^–6^)	0.826	0.349 (0.202)

IBD test was based on the regression of *F*_*st*_/(1 − *F*_*st*_) = *a* + *b* ln(geographical distance), where *a* is the intercept and *b* is the regression coefficient.

**Table 4:** Estimates of parameters for mating system in six natural populations of *Toona ciliata*.

Parameters	Baoshan	Simao	Yongren	Tianlin	Guanshan	Nanping
*t*_*m*_	1.166 (0.067)	0.992 (0.088)	1.082 (0.089)	1.194 (0.031)	0.980 (0.225)	0.843 (0.033)
*α*_*m*_	− 0.166 (0.067)	0.008 (0.088)	− 0.082 (0.090)	− 0.194 (0.031)	0.020 (0.212)	0.157 (0.033)
*t*_*s*_	0.992 (0.038)	0.844 (0.044)	0.899 (0.046)	0.897 (0.022)	0.850 (0.050)	0.672 (0.039)
*α*_*s*_	0.008 (0.038)	0.156 (0.044)	0.100 (0.047)	0.103 (0.022)	0.150 (0.050)	0.338 (0.039)
*t*_*m *_*− t*_*s*_	0.174 (0.085)	0.148 (0.081)	0.183 (0.089)	0.297 (0.041)	0.136 (0.180)	0.171 (0.022)
*F* (maternal)	− 0.187 (0.029)	− 0.178 (0.052)	− 0.141 (0.082)	− 0.184 (0.031)	− 0.027 (0.091)	− 0.200 (0.001)
*r*_*s*_	− 0.765 (0.630)	− 0.546 (0.674)	− 0.292 (0.765)	− 0.919 (0.391)	− 0.261 (0.789)	0.016 (0.048)
*r*_*p(m)*_	0.109 (0.037)	− 0.133 (0.274)	0.080 (0.109)	0.208 (0.055)	0.304 (0.120)	0.312 (0.060)
*r*_*p(s)*_	0.069 (0.054)	− 0.083 (0.180)	0.116 (0.156)	0.281 (0.121)	0.350 (0.184)	0.452 (0.087)
*r*_*p(s) *_*− r*_*p(m)*_	− 0.039 (0.045)	0.050 (0.282)	0.036 (0.157)	0.073 (0.104)	0.046 (0.187)	0.140 (0.043)
*r*_*loci*_	0.293 (0.572)	− 0.022 (0.238)	− 0.054 (0.283)	− 0.086 (0.107)	0.017 (0.172)	− 0.007 (0.108)

*t*_*m*_: the multilocus outcrossing rate; ${\alpha }_{m}$: the multilocus selfing rate (= 1 − *t*_m_); ${t}_{s}$: the single locus outcrossing rate; ${\alpha }_{s}$ : the single locus selfing rate; *F*: the single locus inbreeding coefficient of maternal parents; ${r}_{s}$: the correlation of selfing among families; ${r}_{p}$: the correlation of paternity among siblings; ${r}_{loci}$: the correlation of selfing among loci.

According to the instruction provided in MLTR^18^, null alleles at relevant loci in different populations were counted in estimating the outcrossing rate (or the selfing rate). Results showed that all populations exhibited a predominant outcrossing system (Table 4). Estimates of the multilocus outcrossing rates ranged from 0.843 (Nanping) to 1.194 (Tianlin), with a mean of 0.970 (0.063) over the six populations [*t*_*m*_ in Baoshan,Yongren and Tianlin was set as 1.0 in calculating the mean and standard deviation]. Estimates of the single outcrossing rates ranged from 0.672 (Nanping) to 0.992 (Baoshan), with the mean of 0.859 (0.106). From the relation of selfing and outcrossing rates, *α* = 1 − *t* and their equal variances between *α* and *t*, significant selfing rates were present in Nanping (*α* = 0.157, Se = 0.033) but not in the remaining five populations for the multilocus estimates. However, significant selfing rates at single loci were present in all populations except Baoshan. Significant differences between *t*_*m*_ and *t*_*s*_ occurred in Nanping and Simao, indicating that biparental inbreeding in addition to the occurrence of selfing in these populations^6^. Inbreeding coefficient of maternal parents (*F*) was not significant from zero in Yongren and Guanshan but were significantly negative in the remaining four populations, indicating no inbreeding in female parents.

The correlation of selfing among families was not significant in all populations, indicating independence among families in selfing. The positive correlation of paternity at multiple loci was significant in Baoshan (*r*_*p(m)*_ = 0.109, Se = 0.037), Tianlin (*r*_*p(m)*_ = 0.208, Se = 0.055), Guanshan (*r*_*p(m)*_ = 0.304, Se = 0.120), and Nanping (*r*_*p(m)*_ = 0.312, Se = 0.06), indicating that the same fathers in pollination were partially shared in these populations but not in Yongren (*r*_*p(m)*_ = 0.080, Se = 0.109) and Simao (*r*_*p(m)*_ = − 0.133, Se = 0.274). The results were different from single-locus estimates where a significant correlation of paternity was found only in Nanping (*r*_*p(s)*_ = 0.452, Se = 0.087). Single locus estimates of paternity were significantly greater than multilocus estimate only in Nanping (*r*_*p(s)*_-*r*_*p(m)*_ = 0.140, Se = 0.043), indicating that population substructure occurred in male similarity between outcrosses in Nanping but not in other populations. Significant correlation of selfing among loci (*r*_*loci*_) was not found in all populations, indicating the independence among the eight SSR loci in paternity correlation.

## Discussion

### Mating system

In this study, we confirmed that *T. ciliata* generally exhibited a predominant outcrossing system, which is consistent with most flower structure showing functionally unisexual flowers^2,3^. However, we observed diverse rates of outcrossing in different regions of natural distribution of the species in South China. These estimates of multilocus outcrossing rates reflect the attributes of reproductive ecology of this species. Pollen dispersal could be driven mainly by insects^2^, with an expectation of predominant outcrossing system. However, significant selfing for single locus estimates in Simao, Yongren, Guanshan and Nanping populations or for multilocus estimates in Nanping suggests that this species is self-compatible when there is limitation of pollen within and among populations (Table 4). This study provides evidence of occurrence of selfing and inbreeding despite the floral development that could facilitate cross-breeding. The mixed distribution of functionally male and female flowers on the same inflorescence enhances self-fertilization.

Estimates of paternity correlations indicated that a certain proportion of full-sibship in the same half-sib progeny occurred in four populations except Simao and Yongren that possessed multiple paternity for pollination. Further, Nanping and Guanshan populations exhibited population substructure in male similarity among outcrosses (*r*_*p*(s)_ > *r*_*p*(m)_). This strongly suggests the occurrence of limited pollen dispersal both within and among populations in different regions of the species distribution. Although maternal parents showed lack of inbreeding (*F* = 0), the number of paternal parents was limited in four out of six populations investigated. This could be partially attributed to the limited numbers of pollen donors within or between populations or to the over exploitation that led to low population density. Reports of low natural regeneration was probably associated with the low quality of seeds due to inbreeding depression^9^.

Biparental inbreeding inferred from a comparison of *t*_*m*_ and *t*_*s*_ suggests the presence of cross-fertilization between genetically close relatives in six populations. This may occur in structured populations or in populations of low density where inbreeding takes place within populations. Restriction of pollen flow in *T. ciliata* reinforces biparental inbreeding. Further, distinct pollen pools in allele frequency existed among maternal parents and the progeny so produced came from a few individuals, which could also be inferred from the inverse values of estimates (1/*r*_*p*(m)_) that ranged from 3 to 12 individuals^19^.

The mating system of *T. ciliata* is essentially different from *T. sinensis*, one genetically related species in monophyly genus *Toona* in Meliaceae^1,20^. The differences between *T. ciliata* and *T. sinensis* are subtle in flower morphological traits. The flower structure of *T. sinensis* is characterized by flowers of 4–5 mm long, short pedicels, 5-dentate or shallowly undulate calyxes, five white, oblong and glabrous petals each 4–5 mm long and 2-3 mm wide, ten stamens (five fertile and five degenerate), eight ovules per locule, the style longer than the ovary, and the discoid stigmas with five fine lines. *T. sinensis* is sexually diecious and has the system of complete outcrossing. *T. ciliata* retains perfect flowers, and its mating system is evidently predominant outcrossing from this study. Their qualitative difference in mating system is likely related to the speciation time between *T. ciliata* and *T. sinensis*^21,22^, and distinct evolutionary history modulates each species (inbreeding depression vs. sexual polymorphism).

### Sample sizes and the number of loci

Sampling of seeds is difficult in this species because most mature trees are tall. Rate of seed germination was low in either our green house (South China Agricultural University, Guangzhou) or in the field^23^, resulting in different sizes of progeny populations available for analysis. Although the sample sizes per progeny (4–10 seeds per family) were relatively small in comparison with some reports in the literature, these samples were appropriate for scoring this predominant outcrossing system. These are based on two reasons. One is that microsatellite markers are highly polymorphic and the number of eight loci is sufficient to identify cross individuals. The maternal genotypes can be directly observed (see “Supplemetary materials” for genotyping data) without sophisticated statistical inferences.

The second reason is based on a theoretical study on the array of open-pollinated progeny genotypes descended from maternal parents of unknown genotypes. Simulation studies showed that the optimal number of progeny per family was from 4 to 8 zygotes for estimating fixation index and selfing parameters^24^. This range of sample sizes provides appropriate estimates of variances. The sampling errors are calculated from the binomial distribution by $\sqrt{\widehat{t}(1-\widehat{t})}/n$^18^. Our estimates of standard errors for the outcrossing rate or the selfing rate were less than 10% except Guanshan for the estimate of multilocus outcrossing rate (Table 4).

The number of eight SSR loci was sufficient to identify heterozygotes. This can be viewed from a simple inference. Suppose that all alleles at a locus follow uniform distribution. According to the number of observed alleles at each locus, the joint probability that two individuals had the exact same genotypes at eight loci was infinitely small by chance $\left( { = \frac{1}{14} \cdot \frac{1}{44} \cdot \frac{1}{30} \cdot \frac{1}{30} \cdot \frac{1}{5} \cdot \frac{1}{24} \cdot \frac{1}{47} \cdot \frac{1}{14}} \right)$. Although this probability varied with populations, the power for fingerprinting indidivuals or identifying heterozygotes was large with the eight polymorphic loci.

### Effects of IBD on mating system

Mating system of a natural plant population can be shaped by many factors, including local population density, pollen dispersal within and between populations, plant height, phenological period, and global changes (rapid changes in climate, habitat fragmentation and land usage)^25,26^. With a reference to the information derived from the present analysis of population genetic structure, we here discuss the effects of IBD and gene flow on mating system. Significant IBD effects could be consistent with the entomophilous feature of the species. The relationship between IBD effects within a continuous population and maing system has been long appreciated in theory^27^, and was recently assessed with genome-wide single nucleotide polymorphisms^28^. A relatively higher level of selfing and inbreeding occurred in the eastern part of species distribution, while a lower level of selfing or outcrossing was present in the western part. Pollen flow of *T. ciliata* could be mainly entomophilous, and seed dispersal is mainly mediated by wind together with birds^9^, with an anticipation of a certain level of population genetic differentiation. Previous studies demonstrated that population differentiation of *T*. *ciliata* was substantially large, with *F*_*st*_ = 0.792 among range-wide populations assayed with nuclear SRAP^13^ and 0.324 with nuclear SSR loci among 29 populations^4^. The present study also indicated significant genetic differentiation (*F*_*st*_ = 0.345) among the six populations, and detected significant IBD effects among the six populations. Zhan et al.^4^ showed that IBD effects were absent among populations in the western part but present in the eastern part. IBD effects among populations could likely affect mating system since mating systems differed in different regions under significant IBD effects^29^.

The relationship between IBD between populations and mating system could be complicated^30,31^. From Zhang et al.^31^, an analytical relationship among IBD effects, gene flow and mating system can be roughly inferred by synthesizing the results of Rousset^32^ (*F*_*st*_/(1 − *F*_*st*_) = *a* + *b* ln(geographical distance)), Caballero and Hill^33^ , and Hu and Ennos^34^ under classical island model^35^, i.e.

1 $$ \frac{1}{{N\left. {\left( {1 - \alpha /2} \right)\left( {m_{S} + \left( {1 - \alpha } \right)m_{P} /2} \right)} \right)}} = a + b ln\left( {\text{geographical distance}} \right) $$

where $N\left(1-\alpha /2\right)$ is the effective population size (${N}_{e}$) in which *N* is the population size, *α* is the selfing rate, and *m*_*S*_ and *m*_*P*_ are the migration rates of seeds and pollen, respectively; *a* is the intercept of regression analysis, and *b* is the regression coefficient. A positive correlation is possible between selfing rate (*α*) and geographical distance under significant IBD (*b* > 0) effects.

Thus, two processes could relate IBD effects to mating system. One is the demographic change owing to the reduction of effective population size by selfing/inbreeding, *N* (1 − *α*/2). This change comes from alternation of gene flow within rather than between populations. IBD at the population level reinforces this process by increasing the chance of interbreeding of relatives, which indirectly decreases genetic diversity within populations (e.g., a high level of inbreeding in Nanping population). Reduction in effective population size was reported in an isolated population of *Myracrodruon urundeuva* although pollen dispersal of this species is mediated by wind and bees^36^. The second process is that IBD impedes both seed and pollen flow between rather than within populations, *m*_*S*_ + (1 − *α*) *m*_*P*_/2. Selfing only interacts with pollen flow that is restricted by IBD through decreasing population connectivity or increasing physical distances due to clear-cutting. Empirical studies support the impedement to gene flow by habitat fragmentation^37^ or IBD at the population level, with a few exceptions of case studies^38,39^. Taken together, the interaction of the two processes could reinforce the effects of IBD on mating system.

Concerned with our results (Table 4), the relatively higher rate of selfing or inbreeding in Nanping(*α*_m_ > 0 and *t*_m _− *t*_s_ > 0) and Guanshan (*t*_m _− *t*_s_ > 0) could be associated with Wuyi (2158 m above sea level at the highest peak) and Xuefeng (a part of Yunnan-Guizhou Plateau) Mountains that naturally impeded seed and pollen flow between western and eastern parts of the species distribution (Fig. 1). Parents were mainly confined to local neighbor relatives. Seeds of *T. ciliata* are mainly dispersed by gravity and less often by rodents and birds^9^, and natural regeneration is derived from seeds distributed around mother trees, increasing the rate of both selfing and biparental inbreeding. The average germination rate was about 38% for families^23^, and might reflect inbreeding depression in this species^8^.

### Management implications

Practices of forest management frequently affect population genetic structure within and/or between populations. For instance, selective logging, such as removal of individuals in adults or in the reproductive stage, results in low population density^40^. Other practices of forest management, such as clear-cutting and habitat fragmentation, more or less create population structure^41^. This was the same situation in *T. ciliata* in China where adult trees of straight stem were cut for furniture and natural regeneration by seedlings was low^8^. Carneiro et al.^14^ showed that selective logging in a population of *Hymenaea courbaril* reduced the migration (*m*_*P*_) of pollen from outside populations but increased pollen or seed dispersal distance within the population. They also showed that selective logging reduced the reproductive population size, and increased bottleneck effects (related to *N*_e_) and the rate of selfing or inbreeding among relatives. Conclusions similar to Carneiro et al.^14^ were also drawn in *Baillonella toxisperma*^42^ and other species^41^. Thus, population genetic structure could be affected by selective logging in natural forests of *T. ciliata*^8,9^.

The existence of self-ferilization and inbreeding in *T. ciliata* could produce substantial population genetic differentiation. Management , such as selective logging and clear-cutting followed by natural ecological restoration could yield interbreeding among relatives^14,43^. These practices of conventional forest management erode genetic diversity and destroy habitats. Barriers to the inter-population gene flow enhance population genetic differentiation and reduce genetic variation within populations, leading to high homozygosity in local habitats. This may temporally improve adaptation of subdivided populations to local habitats (high frequency of adaptive homozygotes), but reduce the potential for long-term evolutionary response to globally changing environments. Further, when locally adapted populations hybridize, progeny so produced becomes less fit to local habits, analogous to the outbreeding depression. Therefore, silvicultural management, such as assisted migration of seeds from distinct provenances^44,45^ and maintenance of non-isolated populations in multiple preserved areas, is the key to the viability of *T. ciliata* in China. Since *T. ciliata* is evidently self-compatible, practices that promote extensive pollen dispersal within and between populations, such as improving population density by planting seedlings in logging gaps^42,46^ and/or plantation with genotypes differing from neighborhood trees, can enhance outcrossing and reduce inbreeding depression.

## Conclusions

*T. ciliata* is an important timber species that has been over exploited owing to its high quality of wood for furniture in China. Scoring its mating system helps to understand the reproductive ecology of this species and hence to imply its natural regeneration. This study investigated mating system of six natural populations in different regions and showed occurrence of selfing or inbreeding in different geographical regions. *T. ciliata* exhibited a predominant outcrossing, but allowed selfing and inbreeding. Analysis of population genetic structure showed that substantial genetic variation existed among the six populations. Isolation-by-distance effects were significant among the six populations but insignificant among the four populations in the western part of the distribution of this species. The results suggest that one practice of management is to remove barriers to gene flow in this species, including assisted migration and planting seedlings in logging gaps. The results also indicate the needs of avoiding inbreeding in tree breeding programmes.

## Materials and methods

### Plant material

Six natural populations were selected in 2017 and 2018 (Fig. 1). Three populations were in Yunnan Province (Baoshan, Simao and Yongren), one population in Guangxi Province (Tianlin), one in Jiangxi Province (Guanshan), and one in Fujian Province (Nanping). Samples from Yunnan Province were collected at the altitude of 600–1580 m above sea level in the western part of the species distribution. Samples from Tianlin was collected at 1200 m above sea level in the central region. Samples in Guanshan and Nanping were collected respectively at the altitude of 300 m and 800 m in the eastern part (Table 2). Note that these six populations were resampled and different from those populations published by Zhan et al.^4^ where seed samples were mixed and the pedigree information was not recorded.

Effective sample sizes ranged from 64 in Simao (12 open-pollinated families) to 300 seeds in Nanping (30 open-pollinated families), and an average of 140 seedlings per population (4–10 seeds per family) were germinated for mating system analysis. Note that the sample sizes were appropriate when highly polymorphic markers were employed (say, 50 seeds)^2,23^ and maternal genotypes were observable from progeny genotypes. Seeds were sowed in small containers with mixture of matrix and perlite sand in October of 2017 and 2018 in green house at South China Agricultural University, Guangzhou. Seedlings were grown for 90 days to a height of 2–3 cm.

### DNA extraction and SSR-PCR amplification

DNA extraction was conducted by using 100 mg fresh leaves and following manufacturer’s instructions of the DNAsecure Plant Kit (TIANGEN Biotech Beijing Co., Ltd.). Agarose gel electrophoresis (1.0% w/v) was applied to check the quality of DNA extraction. The DNA concentration was checked with a Thermo Scientific NanoDrop 1000 spectrophotometer (Thermo Fisher Scientific, Waltham, MA, USA), and adjusted to 50 ng μL^−1^. Quantified DNA samples were stored at − 20 °C for PCR amplification.

Selection of SSR markers was based on a previous study of *T. ciliata*^4^. Eight pairs of SSR primers were screened according to the most sequence variability observed in six populations. These SSR markers are nuclear genomes-based, and the resultant amplicons follow biparental Mendelian inheritance. Table 1 shows the SSR primer sequences, repeat motifs, annealing temperature (T_a_), and the observed allele sizes for each SSR locus.

The same procedure as our previous study on *Machilus pauhoi*^47^ was employed. The PCR amplification was carried out in a 25 μL reaction volume containing 1U Taq DNA polymerase (TaKaRa), 1 μL 20 ng μL^−1^ template DNA, 2 μL 25 mmol L^−1^MgCl_2_, 0.5 μL 10 mmol L^−1^dNTP, 0.5 μL 10 mmol L^−1^ of each forward and reverse fluorescent primer pair, 2.5 μL 10× PCR buffer (withoutMgCl2, 100 mmol L^−1^ Tris–HCl, pH 8.8 at 25 °C, 500 mmol L^−1^ KCl). PCR amplification was conducted using following cycle procedure: an initial 3 min of denaturing at 95 °C, followed by ten cycles of three steps (30 s of denaturing at 95 °C, 30 s of annealing at 60 °C, and 30 s of elongation at 72 °C). The next 20 cycles were then used with the same steps (30 s of denaturing at 95 °C, 30 s of annealing at 55 °C, and 30 s of elongation at 72 °C). A final elongation step was at 72 °C for 6 min. The PCR products were detected by capillary electrophoresis using an ABI 3730XL DNA analyzer after confirmation of PCR amplification on a 2% agarose gel. Genemapper 4.0 software (Applied Biosystems) was used to estimate molecular sizes of SSR-PCR amplifications.

### Data analysis

To check genotyping errors, we applied Micro-Checker^17^ to discriminate errors arising from short allele dominance (large allele dropout), stuttering and null alleles. A pooled population consisting of all families was used to check genotyping errors. In this program, presence of null alleles was suggested when homozygotes were overall excess and evenly distributed across the homozygote-classes. Mis-scoring of stutters was suggested when heterozygotes were deficit with alleles differing in size by a single repeat and large homozygotes were in excess. Short allele dominance was suggested when homozygotes were biased towards either extreme of the allele size-distribution. Null allele frequencies were estimated using the method of Oosterhout et al.^17^.

Let ${p}_{ijk}$ be the frequency of allele *k* of locus *j* at population *i*. The expected heterozygosity (*H*_e_) at a locus in a population was calculated by $1-\sum_{k}{p}_{ijk}^{2}$ where the null allele frequency was included. Estimates of allele frequencies were shown in SI Table 2. Following Wright^35^ and Weir^48^, population genetic differentiation at a single locus was calaulated by

2 $${F}_{st(s)}=\frac{\sum_{i}\sum_{k}{n}_{i}{\left({p}_{ijk}-{\stackrel{-}{p}}_{jk}\right)}^{2}/(r-1)\stackrel{-}{n}}{\sum_{k}{\stackrel{-}{p}}_{jk}(1-{\stackrel{-}{p}}_{jk})},$$

where ${\stackrel{-}{p}}_{jk}$ be the average frequency of allele *k* at locus *j* over *r* popuations, and $\stackrel{-}{n}=\sum_{i}{n}_{i}/r$. Test of the null hypothesis H_0_: ${F}_{st(s)}=0$ can be approximated by Chi-square statistic, ${\chi }^{2}=(r-1)\stackrel{-}{n}{F}_{st(s)}$, with *df* = *r *− 1^48^. For multilocus estimate,

3 $${F}_{st(m)}=\frac{\sum_{i}\sum_{j}\sum_{k}{n}_{i}{\left({p}_{ijk}-{\stackrel{-}{p}}_{ijk}\right)}^{2}/(r-1)\stackrel{-}{n}}{\sum_{j}\sum_{k}{\stackrel{-}{p}}_{jk}(1-{\stackrel{-}{p}}_{jk})}.$$

A program in R was provided to calculate population genetic differentiation based on allele frequencies (“SI Program”). Since geographical coordinates of mother trees were not recorded in natural populations, further estimation of contemporary pollen dispersal was not analyzed using POLDISP 1.0^49^.

MLTR was used to handle the presence of null alleles with SSR markers^6,24^ where each allele status at each locus (null allele present with code 1, or absent with code 0) was indicated in preparing dataset. Estimates (expectation–maximization method) of mating system parameters included the multilocus population outcrossing rate (*t*_*m*_), the average single-locus population outcrossing rate (*t*_*s*_), the inbreeding coefficient of maternal parents (*F*), the correlation of paternity at multilocus (*r*_*p*(m)_) and single locus (*r*_*p*(s)_), the correlated selfing rate (*r*_s_), the difference between *t*_m_ and *t*_s_, and the difference between *r*_*p*(s)_ and *r*_*p*(m)_. Because estimates of allele frequencies between pollen and ovules were heterogenous in some half-sib families, their inequality was set in running the program (not pooled), which yielded greater maximum likelihoods with our datasets. Standard errors of population estimates were calculated using 1000 bootstraps that were based on resampling of progeny array.

## Supplementary information

Supplementary Information 1.

Supplementary Information 2.

Supplementary Table 1.
