# Supplementary material for: Mating system and population structure in the natural distribution of Toona ciliata (Meliaceae) in South China
Source: Sci Rep. 2020 Oct 12;10:16998. doi: 10.1038/s41598-020-74123-8 (PMC7550595; doi:10.1038/s41598-020-74123-8)
Supplement: Supplementary file 1 — Supplementary Information 1. [file 41598_2020_74123_MOESM1_ESM.docx]

**Supplementary information**

Genotyping data, allele frequencies estimated in six populations and R program for calculating Fst are provided: Genotyping_data_2020.xls, Suppl_infor_allele_freq_2020.xls, Suppl_Fst_calculation_Rprogram
