# Supplementary material for: Mating system and population structure in the natural distribution of Toona ciliata (Meliaceae) in South China
Source: Sci Rep. 2020 Oct 12;10:16998. doi: 10.1038/s41598-020-74123-8 (PMC7550595; doi:10.1038/s41598-020-74123-8)
Supplement: Supplementary file 2 — Supplementary Information 2. [file 41598_2020_74123_MOESM2_ESM.pdf]

Mating system and population structure in the natural distribution of *Toona ciliata* (Meliaceae) in South China

Wei Zhou [1,2#], Xin-Xin Zhang [1,2#], Ying Ren [1,2], Pei Li [1,2], Xiao-Yang Chen [1,2*], Xin-Sheng Hu [1,2 *]

1 College of Forestry and Landscape Architecture, South China Agricultural University, Guangdong 510642, China

2 Guangdong Key Laboratory for Innovative Development and Utilization of Forest Plant Germplasm, South China Agricultural University, Guangdong 510642, China

#: Contribute to this work equally.

*Corresponding authors: xychen@scau.edu.cn; xinsheng@scau.edu.cn

Supplementary Information: R program for calculating Fst based on allele frequencies

Program function

```
function(infile0, infile){

smpl=array();
smpl<-infile0;

locusid=array();
locusnmb=array();
popsample=array();
kk=numeric();
i=numeric();
j=numeric();

popsample<-names(infile);

indnum=numeric();
indnum<-length(popsample);

poplst=list();
popid1=array();
popid2=array();

yy=array();
aa=character();

for(i in 1:indnum){
   aa<-paste("infile$",popsample[i],sep="");
   yy<-eval(parse(text=aa));
   poplst[[i]]<-yy;
                  }
nn<-length(poplst[[1]]);
k=numeric();

kk=0;

locusid<-poplst[[1]];
kk=1;
k=1;
for(i in 2:nn){
   if(locusid[i]==locusid[i-1]){
     k<-k+1;
    }
   if(locusid[i]!=locusid[i-1]){
     locusnmb[kk]=k;
     kk=kk+1;
     k=1;}
    if(i==nn){locusnmb[kk]=k;}
         }
k0=numeric();
p1=numeric();
p2=numeric();
T1=numeric();
```

```
T2=numeric();
pA1=numeric();
pA2=numeric();
nbar=numeric();
id1=array();
id2=array();
fst=array();
kz=numeric();
kz=0;
m=numeric();
kk0=numeric();
locus=array();

# Fst pairwise populations/per-locus
kk0=0;
for(i in 1:kk){
   p1=0;p2=0;k=0;
   for(p1 in 2:(indnum-1)){
       k=p1+1;
       for(p2 in k:indnum){
          T1=0.0;
          T2=0.0;
          if(i==1) {kk0=1;}
          if(i>1){kk0=locusnmb[i-1]+1;}
          k0=kk0-1+locusnmb[i];
          pA1=0.0;pA2=0.0;nbar=0.0;m=0.0;
          for(j in kk0:k0){
             if((poplst[[p1]][j]!=0.0)|(poplst[[p2]][j]!=0.0)){
                pA1=poplst[[p1]][j];
                pA2=poplst[[p2]][j];
                m=(pA1*smpl[p1-1]+pA2*smpl[p2-1])/(smpl[p1-1]+smpl[p2-1]);

                nbar=(smpl[p1-1]+smpl[p2-1])/2;
                T1=T1+smpl[p1-1]*(pA1-m)*(pA1-m)/nbar+smpl[p2-1]*(pA2-m)*(pA2-m)/nbar;
                T2=T2+m*(1-m);
                   }
               }
          kz=kz+1;
          locus[kz]=i;
          id1[kz]=p1-1;
          id2[kz]=p2-1;
          fst[kz]=T1/T2;
                       } #loop p2
                      } #loop p1
   } #loop i

#Fst pairwise populations /multilocus
for(p1 in 2:(indnum-1)){
    k=p1+1;
    for(p2 in k:indnum){
      T1=0.0;
      T2=0.0;
      for(i in 1:kk){
          if(i==1) {kk0=1;}
          if(i>1){kk0=locusnmb[i-1]+1;}
          k0=kk0-1+locusnmb[i];
```

```
      pA1=0.0;pA2=0.0;nbar=0.0;m=0.0;
      for(j in kk0:k0){
         if((poplst[[p1]][j]!=0.0)|(poplst[[p2]][j]!=0.0)){
            pA1=poplst[[p1]][j];
            pA2=poplst[[p2]][j];
            m=(pA1*smpl[p1-1]+pA2*smpl[p2-1])/(smpl[p1-1]+smpl[p2-1]);
            nbar=(smpl[p1-1]+smpl[p2-1])/2;
            T1=T1+smpl[p1-1]*(pA1-m)*(pA1-m)/nbar+smpl[p2-1]*(pA2-m)*(pA2-m)/nbar;
            T2=T2+m*(1-m);
               }
         }
               } # loop i

     kz=kz+1;
      locus[kz]=99;
      id1[kz]=p1-1;
      id2[kz]=p2-1;
      fst[kz]=T1/T2;
                 } #loop p2
               } #loop p1
```

# Fst among all populations/perLocus

```
nbar=0.0;
nbar=sum(smpl)/length(smpl);
for(i in 1:kk){
   T1=0.0;
   T2=0.0;
   if(i==1) {kk0=1;}
   if(i>1){kk0=locusnmb[i-1]+1;}
   k0=kk0-1+locusnmb[i];
   for(j in kk0:k0){
      m=0.0;
      for(p1 in 2:indnum){ m=m+smpl[p1-1]*poplst[[p1]][j];}
      m=m/sum(smpl);
      for(p1 in 2:indnum){T1=T1+smpl[p1-1]*(poplst[[p1]][j]-m)*(poplst[[p1]][j]-m)/((length(smpl)-1)*nbar);}
      T2=T2+m*(1-m);
            }
      kz=kz+1;
      locus[kz]=i;
      id1[kz]=99;
      id2[kz]=99;
      fst[kz]=T1/T2;
         } #loop i
```

# Fst among all populations/multiLocus

```
nbar=0.0;
nbar=sum(smpl)/length(smpl);
T1=0.0;
T2=0.0;
for(i in 1:kk){
   if(i==1) {kk0=1;}
   if(i>1){kk0=locusnmb[i-1]+1;}
   k0=kk0-1+locusnmb[i];
   for(j in kk0:k0){
      m=0.0;
      for(p1 in 2:indnum){ m=m+smpl[p1-1]*poplst[[p1]][j];}
```

```
    m=m/sum(smpl);
    for(p1 in 2:indnum){T1=T1+smpl[p1-1]*(poplst[[p1]][j]-m)*(poplst[[p1]][j]-m)/((length(smpl)-1)*nbar);}
    T2=T2+m*(1-m);
            }
        }#loop i
    kz=kz+1;
    locus[kz]=99;
    id1[kz]=99;
    id2[kz]=99;
    fst[kz]=T1/T2;

return(data. frame(locus,id1,id2,fst));

}
```

Data Format for Input

infile0<-c(132, 330, 119, 64, 90, 300)

"**inflie0**" records the sample sizes corresponding to populations in "**freqdat.csv**".
The infile is "**freqdat.csv**" given below:

ID is the locus id code: 1-TCR17, 2-TCR18, 3-TCR20, 4-TCR26, 5-TCR51, 6-TCR78, 7-TCR83, 8-TCR122

"**freqdat.csv**" file:

```
ID,Baoshan,Guanshan,Tianlin,Simao,Yongren,Nanping
1,0,0,0,0.0399,0,0
1,0,0,0,0,0.0056,0
1,0.011,0,0.026,0.0318,0.0691,0
1,0.678,0.004,0.3938,0.75,0.6199,0
1,0,0,0.1783,0,0.0056,0
1,0.004,0,0,0,0,0
1,0.004,0,0,0,0,0
1,0.072,0.812,0.0755,0,0.0112,1
1,0,0.029,0.0709,0,0,0
1,0.201,0,0.0755,0,0,0
1,0.03,0.145,0,0,0,0
1,0,0.007,0,0,0,0
1,0,0.004,0,0,0,0
1,0,0,0.18,0.1783,0.2886,0
2,0.004,0,0,0,0,0
2,0,0,0.004,0,0,0
2,0,0,0.004,0,0,0
2,0.008,0,0,0,0.006,0
2,0,0,0.004,0,0,0
2,0.004,0,0,0,0,0
2,0.004,0,0,0,0,0
2,0.622,0,0.585,0.727,0.64,0
2,0.004,0,0.017,0,0,0
2,0,0,0.004,0.008,0,0
2,0,0,0,0.008,0,0
2,0,0,0.009,0,0,0
2,0,0,0.004,0,0,0
2,0,0,0.004,0,0,0
2,0,0,0,0.008,0,0
```

2,0,0,0.004,0,0,0
2,0.008,0,0,0,0,0
2,0.004,0,0.004,0,0.006,0
2,0,0,0,0.006,0
2,0,0,0,0.008,0,0
2,0.004,0,0.004,0,0,0
2,0.004,0,0,0,0,0
2,0,0,0.004,0,0,0
2,0,0,0.004,0,0,0
2,0,0,0,0.008,0,0
2,0,0,0.004,0,0,0
2,0,0,0,0.023,0.006,0.0374
2,0,0,0.013,0,0,0
2,0.126,0.2827,0.226,0.055,0.14,0.3417
2,0.008,0,0.013,0,0,0
2,0,0,0.004,0.023,0.039,0
2,0,0,0.004,0,0,0
2,0.065,0.3921,0.013,0,0.056,0
2,0.019,0,0,0.078,0.073,0
2,0.015,0,0.013,0,0,0
2,0.004,0,0,0.016,0.022,0.2211
2,0,0,0.004,0,0,0
2,0.004,0,0,0.031,0.006,0
2,0.008,0,0,0,0,0
2,0,0,0,0.008,0,0
2,0.008,0,0.009,0,0,0
2,0.065,0,0.038,0,0,0
2,0.015,0,0,0,0,0
2,0,0.3252,0,0,0,0.3998
3,0.004,0,0,0,0,0
3,0.2658,0.0037,0.0762,0,0,0
3,0,0.0037,0,0,0,0
3,0,0.1511,0,0,0,0
3,0.004,0,0,0,0,0
3,0.004,0,0,0,0,0
3,0,0,0.0043,0,0,0
3,0,0.0037,0,0,0,0
3,0,0.0037,0,0,0,0
3,0.004,0,0.0623,0.0318,0.0699,0
3,0.448,0,0.483,0.75,0.7404,0
3,0,0.1775,0.0174,0,0.0113,0
3,0.004,0,0,0,0,0
3,0,0.011,0,0,0,0
3,0,0.0074,0,0,0,0
3,0.004,0,0.0043,0,0,0
3,0,0,0,0,0,0.0185
3,0,0,0.0043,0,0,0
3,0,0.0074,0,0,0,0.2211
3,0,0.0607,0,0,0,0.2084
3,0,0,0.0043,0,0,0
3,0,0.0337,0,0,0,0.0426
3,0,0,0,0,0,0.2362
3,0,0.1555,0,0,0,0
3,0,0.0685,0,0,0,0
3,0.004,0,0,0,0,0
3,0.004,0,0,0,0,0

3,0.004,0,0,0,0,0
3,0,0.1686,0,0,0,0
3,0.2502,0.1438,0.3439,0.2182,0.1784,0.2732
4,0,0.0037,0,0,0,0
4,0,0.8792,0,0,0,0.002
4,0,0.0073,0,0,0,0.49
4,0.012,0,0,0,0,0
4,0,0,0.004,0,0,0
4,0.004,0,0,0,0,0.508
4,0.535,0,0.286,0.352,0.428,0
4,0,0,0,0,0.006,0
4,0.102,0,0.269,0.188,0.211,0
4,0.031,0,0.06,0.125,0,0
4,0,0,0.004,0,0.061,0
4,0.051,0.0037,0.073,0.062,0.006,0
4,0,0,0,0,0.006,0
4,0.098,0.0037,0.094,0.062,0.133,0
4,0,0,0,0,0.056,0
4,0.004,0,0.064,0.016,0,0
4,0,0,0,0,0.011,0
4,0.027,0,0,0.008,0.017,0
4,0.102,0,0.021,0.047,0.011,0
4,0,0,0,0,0.017,0
4,0.012,0,0.004,0.102,0.028,0
4,0.008,0,0.004,0.023,0,0
4,0,0,0.004,0,0,0
4,0.004,0,0.051,0.008,0,0
4,0,0,0.038,0,0,0
4,0.004,0,0,0.008,0,0
4,0,0,0.013,0,0.006,0
4,0.008,0,0.009,0,0,0
4,0,0,0,0,0.006,0
4,0,0.1024,0,0,0,0
5,0.015,0,0.0087,0,0,0
5,0.917,0.996,0.8687,1,1,0.6935
5,0,0,0,0,0,0.1408
5,0.068,0.004,0.013,0,0,0
5,0,0.1096,0,0,0.1657
6,0,0.004,0,0,0,0
6,0.004,0,0,0,0,0
6,0,0.004,0,0,0,0
6,0,0.011,0,0,0,0
6,0.038,0.037,0.096,0.016,0.017,0
6,0,0.015,0,0,0,0
6,0.845,0.111,0.726,0.906,0.883,0
6,0,0.011,0,0,0,0
6,0,0.019,0,0,0,0
6,0,0.007,0,0,0,0
6,0.057,0.004,0.126,0.078,0.083,0
6,0,0,0,0,0,0.579
6,0,0.004,0,0,0,0.0254
6,0.004,0,0,0,0,0
6,0.008,0.015,0.009,0,0.006,0
6,0.038,0.493,0.035,0,0.011,0.306
6,0,0.081,0,0,0,0
6,0,0.004,0,0,0,0

6,0,0.167,0,0,0,0
6,0,0.015,0,0,0,0
6,0.004,0,0,0,0,0
6,0.004,0,0,0,0,0
6,0,0,0.009,0,0,0
6,0,0,0,0,0,0.0896
7,0,0,0,0.0078,0,0
7,0,0,0,0,0.0056,0
7,0,0,0,0.0646,0.0168,0
7,0,0,0,0.0078,0,0
7,0,0,0.004,0,0,0
7,0,0,0,0.0078,0,0
7,0.015,0.779,0.009,0,0,0.193
7,0,0.076,0,0,0,0.037
7,0,0.014,0,0,0,0
7,0.396,0.127,0.397,0.75,0.7892,0.682
7,0.004,0,0,0.0078,0,0
7,0.004,0,0.017,0,0,0
7,0.042,0,0.06,0,0,0
7,0.012,0,0.009,0,0.0282,0
7,0,0.004,0,0,0,0
7,0,0,0.013,0,0,0.088
7,0,0,0.013,0,0,0
7,0,0,0.004,0,0,0
7,0.015,0,0,0,0,0
7,0.012,0,0.004,0,0,0
7,0.004,0,0.004,0,0,0
7,0.015,0,0,0,0,0
7,0.012,0,0.022,0,0.0112,0
7,0.035,0,0.039,0,0.0056,0
7,0.004,0,0,0,0,0
7,0.104,0,0.039,0.0078,0.0225,0
7,0.012,0,0.004,0,0.0112,0
7,0.004,0,0.013,0,0,0
7,0,0,0.004,0,0,0
7,0.05,0,0.009,0,0.0112,0
7,0.019,0,0.004,0,0,0
7,0.054,0,0.086,0,0,0
7,0.004,0,0.009,0,0,0
7,0.104,0,0.159,0,0.0056,0
7,0,0,0.039,0,0.0056,0
7,0,0,0.009,0,0,0
7,0.008,0,0.022,0,0,0
7,0.004,0,0,0,0,0
7,0.004,0,0.004,0,0,0
7,0,0,0.004,0,0,0
7,0.015,0,0,0,0,0
7,0.027,0,0,0,0,0
7,0.004,0,0,0,0.0056,0
7,0.004,0,0,0,0,0
7,0.012,0,0,0,0,0
7,0.004,0,0,0,0,0
7,0,0,0,0.1464,0.0817,0
8,0.004,0,0,0,0,0
8,0,0,0,0.023,0,0
8,0,0,0.004,0,0,0

8,0.723,0.036,0.598,0.938,0.928,0.002
8,0,0,0.004,0,0,0
8,0.061,0.685,0.038,0.039,0.006,0.998
8,0,0.004,0,0,0,0
8,0.004,0,0,0,0.006,0
8,0,0.004,0,0,0,0
8,0,0.004,0,0,0,0
8,0,0,0.004,0,0,0
8,0.201,0,0.346,0,0.061,0
8,0.008,0.261,0,0,0,0
8,0,0.007,0.004,0,0,0

SI Table 2: Estimates of allele frequencies at SSR loci in six populations of Toon ciliata

Loci	Baoshan	Guanshan	Tianlin	Simao	Yongren	Nanping
TCR17						
140	0	0	0	0.0399	0	0
151	0	0	0	0	0.0056	0
152	0.011	0	0.026	0.0318	0.0691	0
153	0.678	0.004	0.3938	0.75	0.6199	0
154	0	0	0.1783	0	0.0056	0
155	0.004	0	0	0	0	0
156	0.004	0	0	0	0	0
158	0.072	0.812	0.0755	0	0.0112	1
159	0	0.029	0.0709	0	0	0
191	0.201	0	0.0755	0	0	0
192	0.03	0.145	0	0	0	0
193	0	0.007	0	0	0	0
205	0	0.004	0	0	0	0
Null allele	0	0	0.18	0.1783	0.2886	0
TCR18						
107	0.004	0	0	0	0	0
117	0	0	0.004	0	0	0
121	0	0	0.004	0	0	0
124	0.008	0	0	0	0.006	0
127	0	0	0.004	0	0	0
128	0.004	0	0	0	0	0
133	0.004	0	0	0	0	0
140	0.622	0	0.585	0.727	0.64	0
142	0.004	0	0.017	0	0	0
143	0	0	0.004	0.008	0	0
144	0	0	0	0.008	0	0
146	0	0	0.009	0	0	0
151	0	0	0.004	0	0	0
152	0	0	0.004	0	0	0
155	0	0	0	0.008	0	0
157	0	0	0.004	0	0	0
158	0.008	0	0	0	0	0
162	0.004	0	0.004	0	0.006	0
163	0	0	0	0	0.006	0
164	0	0	0	0.008	0	0
165	0.004	0	0.004	0	0	0
168	0.004	0	0	0	0	0
171	0	0	0.004	0	0	0
172	0	0	0.004	0	0	0
173	0	0	0	0.008	0	0
174	0	0	0.004	0	0	0
180	0	0	0	0.023	0.006	0.0374
181	0	0	0.013	0	0	0
182	0.126	0.2827	0.226	0.055	0.14	0.3417

183	0.008	0	0.013	0	0	0
184	0	0	0.004	0.023	0.039	0
185	0	0	0.004	0	0	0
186	0.065	0.3921	0.013	0	0.056	0
188	0.019	0	0	0.078	0.073	0
189	0.015	0	0.013	0	0	0
190	0.004	0	0	0.016	0.022	0.2211
191	0	0	0.004	0	0	0
192	0.004	0	0	0.031	0.006	0
193	0.008	0	0	0	0	0
194	0	0	0	0.008	0	0
201	0.008	0	0.009	0	0	0
202	0.065	0	0.038	0	0	0
210	0.015	0	0	0	0	0
Null allele	0	0.3252	0	0	0	0.3998
TCR20						
152	0.004	0	0	0	0	0
153	0.2658	0.0037	0.0762	0	0	0
157	0	0.0037	0	0	0	0
158	0	0.1511	0	0	0	0
251	0.004	0	0	0	0	0
260	0.004	0	0	0	0	0
268	0	0	0.0043	0	0	0
283	0	0.0037	0	0	0	0
285	0	0.0037	0	0	0	0
292	0.004	0	0.0623	0.0318	0.0699	0
293	0.448	0	0.483	0.75	0.7404	0
297	0	0.1775	0.0174	0	0.0113	0
298	0.004	0	0	0	0	0
299	0	0.011	0	0	0	0
300	0	0.0074	0	0	0	0
301	0.004	0	0.0043	0	0	0
303	0	0	0	0	0	0.0185
304	0	0	0.0043	0	0	0
305	0	0.0074	0	0	0	0.2211
307	0	0.0607	0	0	0	0.2084
308	0	0	0.0043	0	0	0
311	0	0.0337	0	0	0	0.0426
313	0	0	0	0	0	0.2362
315	0	0.1555	0	0	0	0
319	0	0.0685	0	0	0	0
322	0.004	0	0	0	0	0
323	0.004	0	0	0	0	0
324	0.004	0	0	0	0	0
327	0	0.1686	0	0	0	0
Null allele	0.2502	0.1438	0.3439	0.2182	0.1784	0.2732
TCR26						

233	0	0.0037	0	0	0	0
234	0	0.8792	0	0	0	0.002
235	0	0.0073	0	0	0	0.49
236	0.012	0	0	0	0	0
238	0	0	0.004	0	0	0
239	0.004	0	0	0	0	0.508
240	0.535	0	0.286	0.352	0.428	0
241	0	0	0	0	0.006	0
242	0.102	0	0.269	0.188	0.211	0
244	0.031	0	0.06	0.125	0	0
245	0	0	0.004	0	0.061	0
246	0.051	0.0037	0.073	0.062	0.006	0
247	0	0	0	0	0.006	0
248	0.098	0.0037	0.094	0.062	0.133	0
249	0	0	0	0	0.056	0
250	0.004	0	0.064	0.016	0	0
251	0	0	0	0	0.011	0
252	0.027	0	0	0.008	0.017	0
254	0.102	0	0.021	0.047	0.011	0
255	0	0	0	0	0.017	0
256	0.012	0	0.004	0.102	0.028	0
258	0.008	0	0.004	0.023	0	0
259	0	0	0.004	0	0	0
260	0.004	0	0.051	0.008	0	0
261	0	0	0.038	0	0	0
262	0.004	0	0	0.008	0	0
264	0	0	0.013	0	0.006	0
265	0.008	0	0.009	0	0	0
271	0	0	0	0	0.006	0
Null allele	0	0.1024	0	0	0	0
TCR51						
111	0.015	0	0.0087	0	0	0
112	0.917	0.996	0.8687	1	1	0.6935
114	0	0	0	0	0	0.1408
140	0.068	0.004	0.013	0	0	0
Null allele	0	0	0.1096	0	0	0.1657
TCR78						
163	0	0.004	0	0	0	0
168	0.004	0	0	0	0	0
169	0	0.004	0	0	0	0
170	0	0.011	0	0	0	0
172	0.038	0.037	0.096	0.016	0.017	0
174	0	0.015	0	0	0	0
176	0.845	0.111	0.726	0.906	0.883	0
177	0	0.011	0	0	0	0
178	0	0.019	0	0	0	0
179	0	0.007	0	0	0	0

180	0.057	0.004	0.126	0.078	0.083	0
181	0	0	0	0	0	0.579
183	0	0.004	0	0	0	0.0254
184	0.004	0	0	0	0	0
186	0.008	0.015	0.009	0	0.006	0
188	0.038	0.493	0.035	0	0.011	0.306
190	0	0.081	0	0	0	0
202	0	0.004	0	0	0	0
226	0	0.167	0	0	0	0
228	0	0.015	0	0	0	0
231	0.004	0	0	0	0	0
252	0.004	0	0	0	0	0
255	0	0	0.009	0	0	0
Null allele	0	0	0	0	0	0.0896
TCR83						
183	0	0	0	0.0078	0	0
186	0	0	0	0	0.0056	0
191	0	0	0	0.0646	0.0168	0
192	0	0	0	0.0078	0	0
200	0	0	0.004	0	0	0
218	0	0	0	0.0078	0	0
226	0.015	0.779	0.009	0	0	0.193
228	0	0.076	0	0	0	0.037
229	0	0.014	0	0	0	0
231	0.396	0.127	0.397	0.75	0.7892	0.682
232	0.004	0	0	0.0078	0	0
234	0.004	0	0.017	0	0	0
240	0.042	0	0.06	0	0	0
241	0.012	0	0.009	0	0.0282	0
242	0	0.004	0	0	0	0
245	0	0	0.013	0	0	0.088
246	0	0	0.013	0	0	0
247	0	0	0.004	0	0	0
248	0.015	0	0	0	0	0
249	0.012	0	0.004	0	0	0
250	0.004	0	0.004	0	0	0
251	0.015	0	0	0	0	0
252	0.012	0	0.022	0	0.0112	0
253	0.035	0	0.039	0	0.0056	0
254	0.004	0	0	0	0	0
255	0.104	0	0.039	0.0078	0.0225	0
256	0.012	0	0.004	0	0.0112	0
257	0.004	0	0.013	0	0	0
258	0	0	0.004	0	0	0
259	0.05	0	0.009	0	0.0112	0
260	0.019	0	0.004	0	0	0
261	0.054	0	0.086	0	0	0

262	0.004	0	0.009	0	0	0
263	0.104	0	0.159	0	0.0056	0
264	0	0	0.039	0	0.0056	0
265	0	0	0.009	0	0	0
267	0.008	0	0.022	0	0	0
269	0.004	0	0	0	0	0
271	0.004	0	0.004	0	0	0
273	0	0	0.004	0	0	0
274	0.015	0	0	0	0	0
275	0.027	0	0	0	0	0
276	0.004	0	0	0	0.0056	0
278	0.004	0	0	0	0	0
286	0.012	0	0	0	0	0
287	0.004	0	0	0	0	0
Null allele				0.1464	0.0817	
TCR122						
158	0.004	0	0	0	0	0
162	0	0	0	0.023	0	0
163	0	0	0.004	0	0	0
164	0.723	0.036	0.598	0.938	0.928	0.002
165	0	0	0.004	0	0	0
166	0.061	0.685	0.038	0.039	0.006	0.998
170	0	0.004	0	0	0	0
172	0.004	0	0	0	0.006	0
180	0	0.004	0	0	0	0
231	0	0.004	0	0	0	0
294	0	0	0.004	0	0	0
295	0.201	0	0.346	0	0.061	0
297	0.008	0.261	0	0	0	0
298	0	0.007	0.004	0	0	0
Null allele	0	0	0	0	0	0
